# Supplementary material for: Porphyrins and Metalloporphyrins Combined with N-Heterocyclic Carbene (NHC) Gold(I) Complexes for Photodynamic Therapy Application: What Is the Weight of the Heavy Atom Effect?
Source: Molecules. 2022 Jun 23;27(13):4046. doi: 10.3390/molecules27134046 (PMC9268150; doi:10.3390/molecules27134046)
Supplement: Supplementary file 1 [file molecules-27-04046-s001.zip › molecules-1772468-supplementary.pdf]

Supporting Information

Porphyrins and Metalloporphyrins Combined with N-heterocyclic Carbenes (NHC) Gold(I) Complexes for Photodynamic Therapy Application. What is the Weight of the Heavy Atom Effect?

Stefano Scoditti [1], Francesco Chiodo,[1] Gloria Mazzone,[1,]* Sébastien Richeter[2] and Emilia Sicilia[1,]*

[1] Department of Chemistry and Chemical Technologies, Università della Calabria, Arcavacata di Rende (CS), 87036, Italy; stefano.scoditti@unical.it, francescochiodo22@gmail.com

[2] ICGM, Univ Montpellier, CNRS, ENSCM, Montpellier 34293, France; sebastien.richeter@umontpellier.fr,

* Correspondence: gloria.mazzone@unical.it, emilia.sicilia@unical.it

Table of Contents

Figure S1: Superimposition of the optimized (green) and crystallographic (silver) structures of **Fused-FbAu**. Two selected computed and crystallographic dihedral angles in degrees are also reported. S2

Table S1: TD-DFT benchmark for **Fused-Fb** and **Fused-FbAu** species optimized at B3LYP/6-31G** level in CH_2Cl_2 implicit solvent. S3

Table S2: M06 maximum wavelengths for the Q and Soret bands calculated for the **Meso-Fb**, **Meso-Zn** and **Meso-ZnAu** species compared with the experimental counterparts. S4

Figure S2: Graphical representation of the highest occupied (HOMO, HOMO-1) and the lowest unoccupied (LUMO, LUMO+1) MOs for **Fused-ZnAu**, **Fused-PdAu** and all the **Meso** compounds. S5

Table S3: Main absorption wavelengths (λ), vertical triplet electronic energies ΔE, oscillator strengths, *f*, and main configuration for the examined **Fused** compounds in water solvent computed at M06/6-31G** level of theory. S6

Table S4: Main absorption wavelengths (λ), vertical triplet electronic energies ΔE, oscillator strengths, *f*, and main configuration for the examined **Meso** compounds in water solvent computed at M06/6-31G** level of theory. S7

Figure S3: Computed singlet-triplet splitting energies, ΔE (eV), between singlet (S_{1-2}) and triplets (T_{1-4}) potentially involved in the ISC for a) **Fused** and b) **Meso** compounds. S8

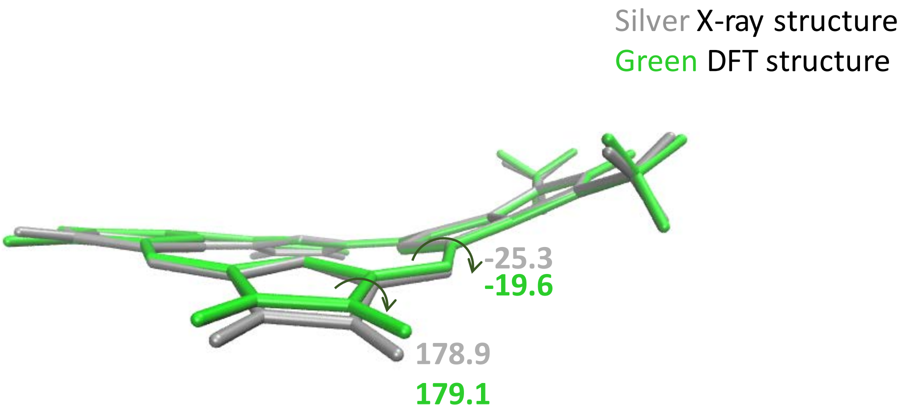

Figure S1: Superimposition of the optimized (green) and crystallographic (silver) structures of **Fused-FbAu**. Two selected computed and crystallographic dihedral angles in degrees are also reported.

Table S1: TD-DFT benchmark for **Fused-Fb** and **Fused-FbAu** species optimized at B3LYP/6-31G** level in CH_2Cl_2 implicit solvent.

**Fused-Fb**	**λ_B(nm)**	***f***	**λ_{Qy}(nm)**	***f***	**λ_{Qx}(nm)**	***f***
λ_{exp} [a](nm)	428		568		657	
B3LYP-D3	414	1.366	556	0.061	594	0.103
PBE0-D3	407	1.720	546	0.061	584	0.100
CAMB3LYP-D3	391	1.768	556	0.032	610	0.066
M06	415	2.052	570	0.046	606	0.091
B3PW91	413	1.966	554	0.066	591	0.104
LC-wPBE	380	1.893	605	0.018	705	0.035
**Fused-FbAu**						
λ_{exp} [b](nm)	429		566		661	
B3LYP-D3	414	2.170	556	0.073	592	0.073
PBE0-D3	407	2.250	545	0.073	582	0.070
CAMB3LYP-D3	393	1.650	556	0.042	609	0.042
M06	416	2.264	570	0.056	603	0.063
B3PW91	413	2.160	554	0.079	589	0.074
LC-wPBE	380	2.510	604	0.0249	708	0.020

[a] Lefebvre, J.F. *European Journal of Organic Chemistry* **2010**, 1912–1920; [b] Longevial, J.F. Organometallics **2016**, 35, 663–672

Table S2: M06 maximum wavelengths for the Q and Soret bands calculated for the **Meso-Fb**, **Meso-Zn** and **Meso-ZnAu** species in the same solvent experimentally used.

Compound	Theo	Exp.[a]
**Meso-Fb**[b]	585	615
	411	413
**Meso-Zn**[c]	557	548
	406	420
**Meso-ZnAu**[c]	558	548
	407	420

a. taken from Rose, C. et al. *Comptes Rendus Chimie* **2021**, *24*. Recorded and computed in b. DMSO or c. CH_2Cl_2

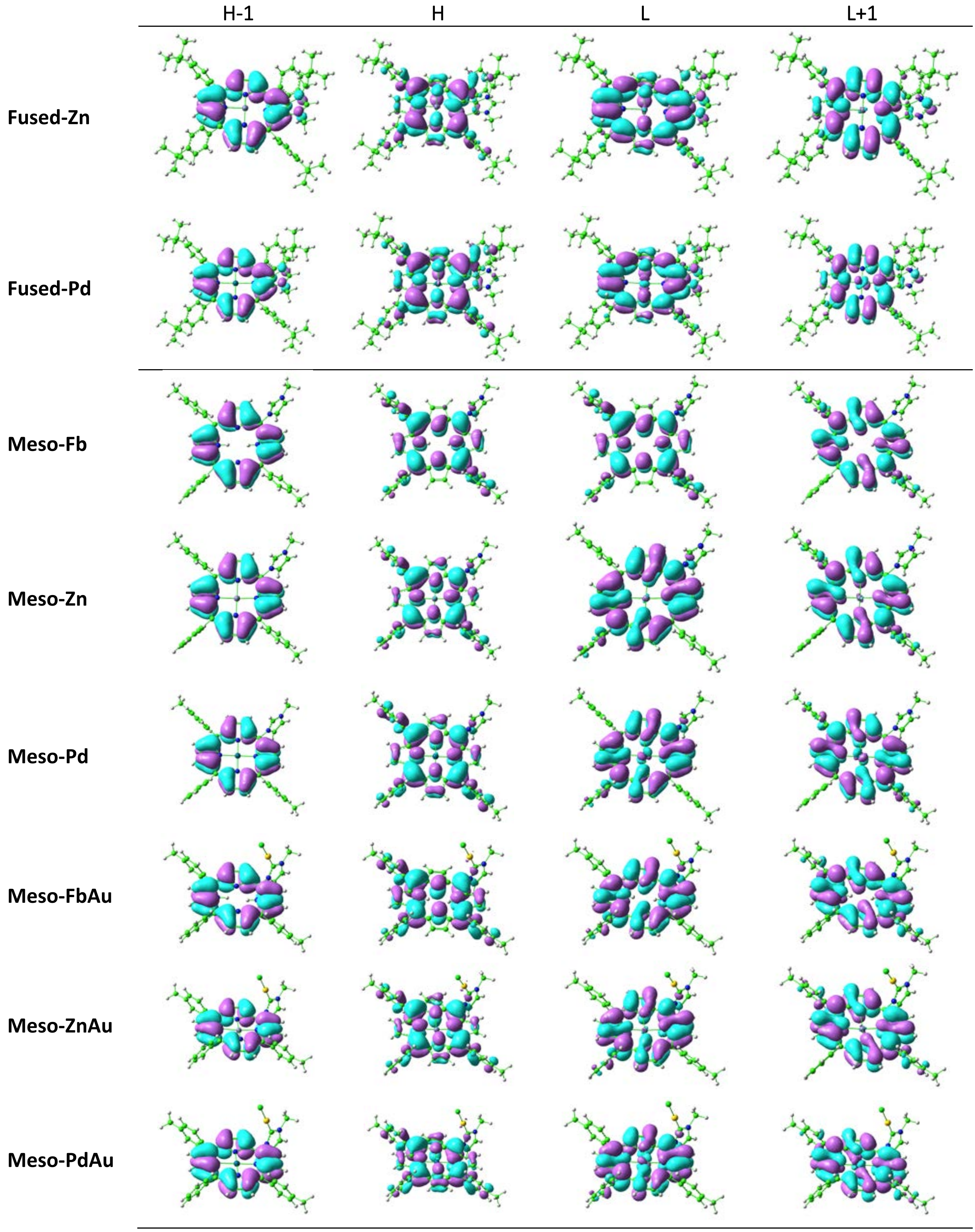

Figure S2: Graphical representation of the highest occupied (H, H-1) and the lowest unoccupied (L, L+1) MOs for **Fused-ZnAu**, **Fused-PdAu** and all the **Meso** compounds.

Table S3: Main absorption wavelengths (λ), vertical triplet electronic energies ΔE, oscillator strengths, *f*, and main configuration for the examined **Fused** compounds in water solvent computed at M06/6-31G** level of theory.

Compound	Excited State	ΔE (eV)	Transitions
**Fused-Fb**	T_1	1.21	H→L+1 (80%), H-1→L (23%)
	T_2	1.50	H→L (96%)
	T_3	1.88	H-1→L (78%), H→L+1 (22%)
	T_4	2.00	H-1→L+1 (96%)
**Fused-Zn**	T_1	1.47	H→L (83%)
	T_2	1.49	H→L+1 (82%)
	T_3	1.94	H-1->LUMO (82%)
	T_4	1.95	H-1->L+1 (83%)
**Fused-Pd**	T_1	1.60	H→L (78%), H-1->L+1 (20%)
	T_2	1.63	H→L+1 (80%)
	T_3	2.04	H-1→L+1 (79%), H→L (20%)
	T_4	2.04	H-1→L (82%)
**Fused-FbAu**	T_1	1.18	H→L+1 (80%), H-1→L (24%)
	T_2	1.54	H→L (96%)
	T_3	1.88	H-1→L (77%), H→L+1 (23%)
	T_4	1.99	H-1->L+1 (96%)
**Fused-ZnAu**	T_1	1.48	H→L (82%)
	T_2	1.49	H→L+1 (81%)
	T_3	1.93	H-1→L (81%)
	T_4	1.94	H-1→L+1 (82%)
**Fused-PdAu**	T_1	1.62	H→L (80%)
	T_2	1.63	H-1→L (22%), H→L+1 (76%)
	T_3	2.02	H-1→L (77%), H→L+1 (23%)
	T_4	2.04	H-1→L+1 (81%)

Table S4: Main absorption wavelengths (λ), vertical triplet electronic energies ΔE, oscillator strengths, *f*, and main configuration for the examined **Meso** compounds in water solvent computed at M06/6-31G** level of theory.

.

Compound	Excited State	ΔE (eV)	Transitions
**Meso-Fb**	T_1	1.26	H→L (41%), H→L+1 (32%), H-1→L (21%)
	T_2	1.63	H→L (52%), H→L+1 (33%)
	T_3	1.89	H-1→L (64%), H→L+1 (35%)
	T_4	1.98	H-1→L+1 (90%)
**Meso-Zn**	T_1	1.52	H→L (86%)
	T_2	1.55	H→L+1 (54%), H-1→L (45%)
	T_3	1.93	H-1→L (55%), H→L+1 (44%)
	T_4	1.99	H-1->L+1 (86%)
**Meso-Pd**	T_1	1.64	H→L (84%)
	T_2	1.67	H→L+1 (50%), H-1→L (48%)
	T_3	2.03	H-1→L (51%), H→L+1 (47%)
	T_4	2.08	H-1->L+1 (85%)
**Meso-FbAu**	T_1	1.23	H→L (41%), H→L+1 (35%)
	T_2	1.63	H→L (52%), H→L+1 (38%)
	T_3	1.89	H-1→L (69%), H→L+1 (27%)
	T_4	1.99	H-1→L+1 (88%)
**Meso-ZnAu**	T_1	1.50	H→L (85%)
	T_2	1.54	H→L+1 (64%), H-1→L (35%)
	T_3	1.93	H-1→L (65%), H→L+1 (35%)
	T_4	1.99	H-1→L+1 (86%)
**Meso-PdAu**	T_1	1.63	H→L (83%)
	T_2	1.67	H→L+1 (60%), H-1→L (38%),
	T_3	2.03	H-1→L (62%), H→L+1 (37%)
	T_4	2.08	H-1→L+1 (84%)

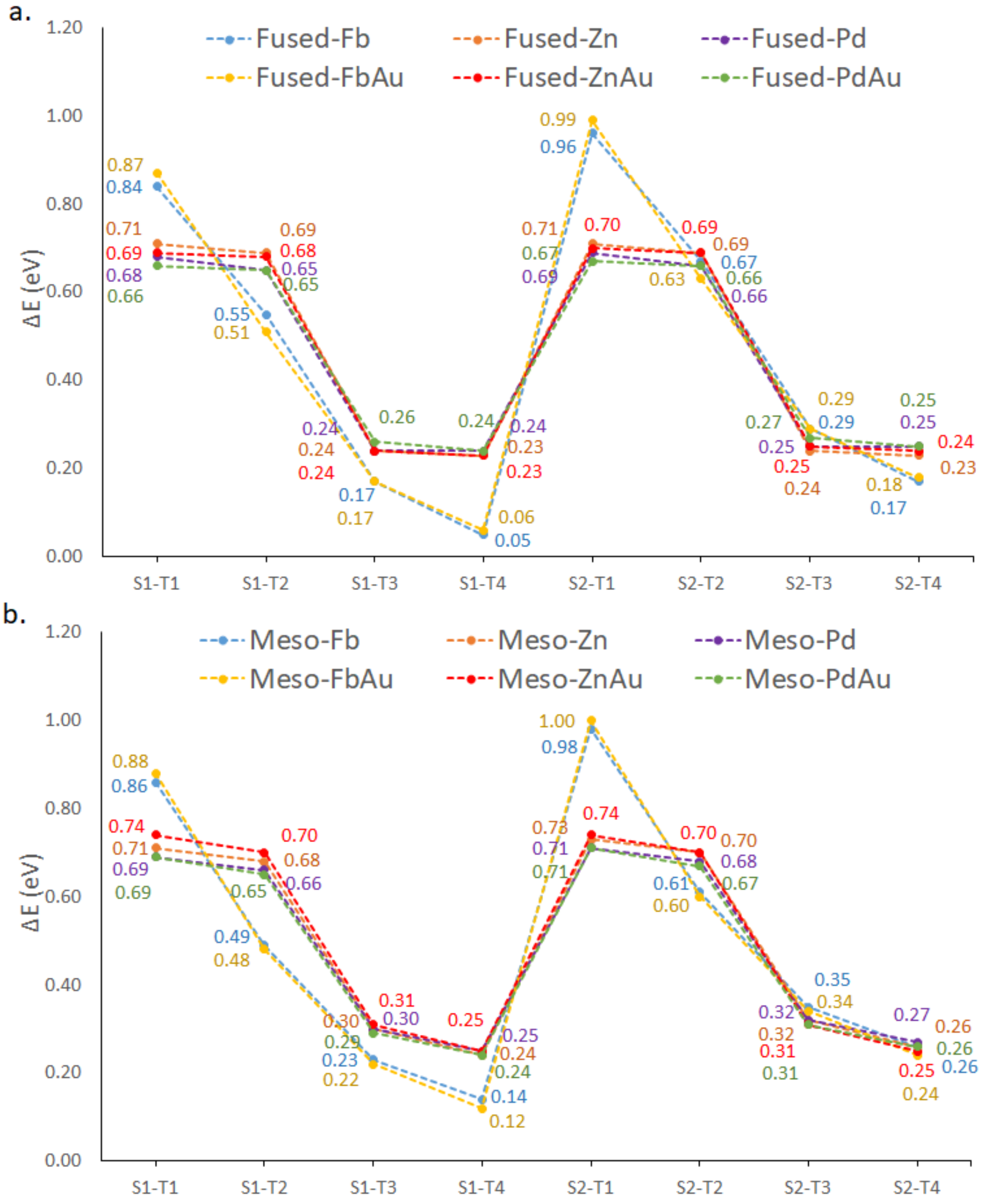

Figure S3: Computed singlet-triplet splitting energies, ΔE (eV), between singlet (S_{1-2}) and triplets (T_{1-4}) potentially involved in the ISC for a) **Fused** and b) **Meso** compounds.
